# Capsule-LPI: a LncRNA–protein interaction predicting tool based on a capsule network

**DOI:** 10.1186/s12859-021-04171-y

**Published:** 2021-05-13

**Authors:** Ying Li, Hang Sun, Shiyao Feng, Qi Zhang, Siyu Han, Wei Du

**Affiliations:** 1 Key Laboratory of Symbolic Computation and Knowledge Engineering, Ministry of Education, College of Computer Science and Technology, Jilin University, Qianjin Street, 130012 Changchun, China; 2 Department of Computer Science, Faculty of Engineering, University of Bristol, Bristol, BS8 1UB UK

**Keywords:** Long noncoding RNA, lncRNA–protein interaction, Capsule network

## Abstract

**Background:**

Long noncoding RNAs (lncRNAs) play important roles in multiple biological processes. Identifying LncRNA–protein interactions (LPIs) is key to understanding lncRNA functions. Although some LPIs computational methods have been developed, the LPIs prediction problem remains challenging. How to integrate multimodal features from more perspectives and build deep learning architectures with better recognition performance have always been the focus of research on LPIs.

**Results:**

We present a novel multichannel capsule network framework to integrate multimodal features for LPI prediction, Capsule-LPI. Capsule-LPI integrates four groups of multimodal features, including sequence features, motif information, physicochemical properties and secondary structure features. Capsule-LPI is composed of four feature-learning subnetworks and one capsule subnetwork. Through comprehensive experimental comparisons and evaluations, we demonstrate that both multimodal features and the architecture of the multichannel capsule network can significantly improve the performance of LPI prediction. The experimental results show that Capsule-LPI performs better than the existing state-of-the-art tools. The precision of Capsule-LPI is 87.3%, which represents a 1.7% improvement. The F-value of Capsule-LPI is 92.2%, which represents a 1.4% improvement.

**Conclusions:**

This study provides a novel and feasible LPI prediction tool based on the integration of multimodal features and a capsule network. A webserver (http://csbg-jlu.site/lpc/predict) is developed to be convenient for users.

**Supplementary Information:**

The online version contains supplementary material available at 10.1186/s12859-021-04171-y.

## Background

Long noncoding RNAs (lncRNAs) are noncoding RNAs that are greater than 200 nt in length and make up the bulk of transcripts [1]. Currently, accumulating research has discovered that lncRNAs play important roles in multiple biological processes [2–5] and are highly associated with diverse human diseases such as tumours and cancers [6–9]. However, the functions and molecular mechanism of the vast majority of lncRNAs remain unknown.

To understand the functions of lncRNAs, there is a fundamental path to identify proteins that interact with lncRNAs. Most lncRNAs need to bind to one or more proteins to function [10]. Based on the LncRNA–protein interaction (LPI) results, further insights into the functions and molecular mechanisms of lncRNAs can be inferred with the help of abundant annotation information of the protein. Therefore, it is of profound significance to study LPIs. There are several ways to explore LPIs, which can be divided into experimental methods [11] and computational methods. Experimental methods are time-consuming and expensive [12], while computational methods are efficient and economical.

There are many computational methods of LPI. For instance, Muppirala et al. developed a computational model called RPISeq [12] in 2011, which applies sequence features of lncRNAs and proteins and contains support vector machine (SVM) and random forests (RF) classifiers. In 2013, Lu et al. proposed a method named LncPro [13], which integrates secondary structure features, hydrogen-bonding propensities, and van der Waals interaction features and chooses matrix computation as the calculation method. Then, Suresh et al. proposed RPI-Pred [14] in 2015, which uses sequence features and structure features to develop a model based on SVM. Later, Akbaripour-Elahabad et al. developed RpiCool [15], which utilizes sequence features and motif features and chooses RF as a classifier. In 2015, Li et al. proposed a novel LPI prediction method LPIHN [16] based on random walks with restart on the heterogeneous network constructed by the lncRNA-lncRNA similarity network, LncRNA–protein interaction network, and protein-protein interaction network. In 2016, a network computational method for LPI prediction on LPBNI [17] was developed by Ge et al. In 2017, Zhang et al. developed LPLNP [18], which integrates interaction profile, expression profile, sequence composition features of lncRNAs and interaction profile, CTD features of proteins, and uses the linear neighbourhood similarity and a label propagation process to predict potential LPI. In the same year, Zhang et al. proposed a sequence-based feature learning method called SFPEL-LPI [19]. SFPEL-LPI uses lncRNA sequences, protein sequences, and known LncRNA–protein interactions to compute three lncRNA-lncRNA similarities and protein-protein similarities and combines them with a feature projection ensemble learning frame. In 2018, Zhao et al. proposed a semisupervised model LPI-BNPRA [20], which integrates the lncRNA similarity matrix, protein similarity matrix, and LncRNA–protein interaction matrix to infer LPI. Then, Zhao et al. developed IRWNRLPI [21] for LncRNA–protein interaction prediction by combining random walk algorithms and neighbourhood regularized logistics, which included the lncRNA similarity matrix, protein similarity matrix, and LncRNA–protein interaction matrix. Hu et al. proposed an ensemble method named HLPI-Ensemble [22] by integrating sequence features and the ensemble strategy based on SVM, RF and eXtreme Gradient Boosting. In 2019, Yi et al. developed LPI-Pred [23], which is inspired by the similarity between natural language and biological sequences. LPI-Pred uses word2vec to obtain RNA2vec and Pro2vec as the word embedding features of lncRNAs and proteins, respectively. RF was selected as a classifier to predict LPI.

In recent years, deep learning models have been used for the prediction of LPI. In 2016, Pan et al. developed the computational method IPMiner [24], which employs sequence features and makes use of deep learning to learn hidden features. Then, three RF models were trained, and stacked ensembling was used to integrate different classifiers to further enhance the prediction performance. In 2018, a comprehensive tool named LncADeep [25] was proposed by Yang et al. In the LPI part, LncADeep integrates the sequence and structure features used in lncPro and some lncRNA features used for lncRNA identification such as Fickett nucleotide features and features of LCDs to infer LPI based on the deep stacking network. In 2020, LPI-CNNCP [26], a novel convolutional neural network method with a copy-padding trick, was proposed by Zhang et al. Zhang et al. also proposed an ensemble deep learning model: lncIBTP [27], which uses sequence features and ensemble CNN and full connection layers as the architecture of lncIBTP. Wekesa et al. proposed a graph representation learning method called GPLPI [28] based on sequence and structural features for LPI prediction. Meanwhile, Wekesa et al. developed a multifeature fusion-based method named DRPLPI [29], which uses a multihead self-attention long short-term memory encoder-decoder network to extract high-level features and feds them into Catboost and extra tree classification algorithms for LPI prediction.

For most application fields, with the support of large sample sets, deep learning models have better learning performance than traditional machine learning. Deep learning architectures are good at high-level feature extraction, which allows end-to-end learning to be implemented. The design of the deep learning architecture is very flexible. Many deep learning architectures such as CNN [30], DBN [31], RNN [32], BiLSTM [33], attention network [34], capsule network [35] and graph neural network [36] have been developed. The capsule network is one of the most representative networks. To improve the performance of LPI prediction and to explore the effectiveness of the capsule network for LPI, a capsule network has been applied for LPI prediction, which is first proposed for the image recognition field. In the image recognition process, multiple depth features obtained by the feature extraction subnetworks can be well used by the capsule network to make predictions [35]. Compared with other deep learning architectures, the capsule network is more sensitive to the relationship between features. One more advantage of the capsule network over other deep learning architectures is that there are very few parameters to be trained. In our architecture, the capsule network part has only 36 parameters that need to be trained, which makes training faster and improves the overfitting.

Inspired by the better feature-learning capability of the capsule network, in addition to capturing the panorama of LPI information, multiple features are combined, including sequence features, motif information, physicochemical properties and secondary structure features. Another reason for using multimodal features is that lncRNAs and proteins are complex and have many aspects such as sequence information, structural information, and physical and chemical information. Single-modal features have difficulty fully representing lncRNA and protein information, so integrating multimodal features can theoretically produce better prediction performance. At the same time, the advantages of the flexible design of deep learning architectures also create opportunities for the use of multimodal features. For example, Deng et al. proposed a multimodal deep learning framework named DDIMDL [37] in 2020, which constructs deep neural network (DNN)-based submodels to deal with four features and then adopts a joint DNN framework to combine the submodels to make a prediction. In recent years, a variety of LPI prediction methods [22, 28, 29] have adopted multimodal features and achieved good results.

Therefore, we propose a novel multichannel capsule network framework to integrate these multimodal features for LPI prediction, capsule-LPI. The main contributions of Capsule-LPI include:

1. Multimodal features are designed to capture the full information of LPI, including sequence features, motif information, physicochemical properties and secondary structure features. More information features such as physicochemical properties and motif information are integrated into Capsule-LPI compared to existing LPI prediction tools.
2. To better integrate and learn multimodal features, a deep learning architecture based on multichannel capsule networks is proposed to integrate the multimodal features.
3. Capsule-LPI outperforms state-of-the-art methods for LPI prediction with a precision of 87.3% and an F-value of 92.2%. Capsule-LPI also has the significant advantage that very few network parameters need to be trained in the feature-binding part, which makes Capsule-LPI require much less time for training and prediction than other deep learning-based tools.
4. To maximize the convenience for users, a webserver (http://csbg-jlu.site/lpc/predict) has been developed. In addition, the source code and dataset used in this paper are provided at http://csbg-jlu.site/lpc/download. The source code usage refers to the “README” file in the source code package.

## Methods

### Capsule-LPI overview

**Fig. 1:**
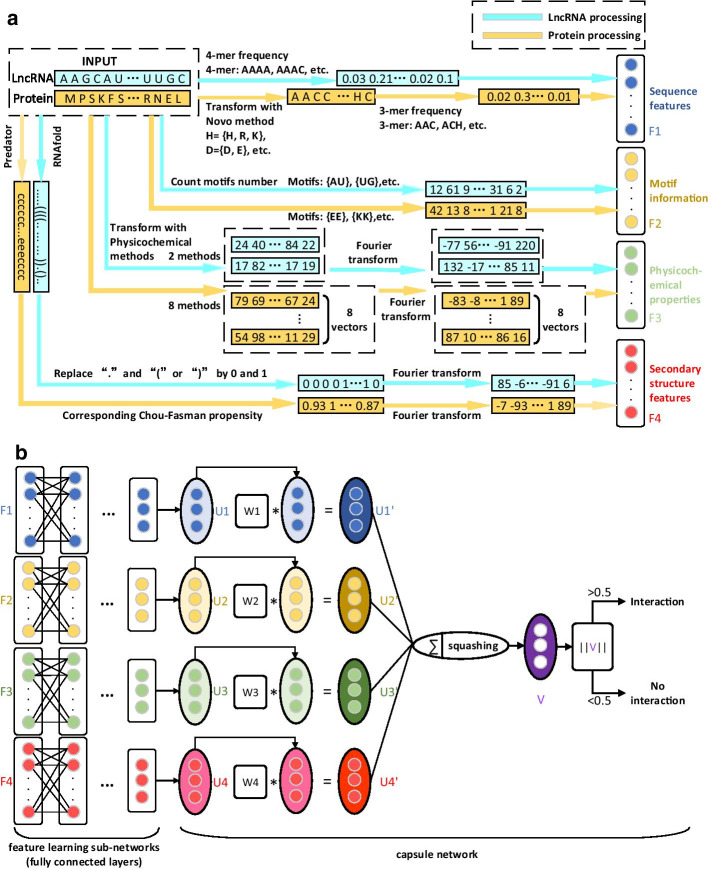
Flowchart of Capsule-LPI. **a** Multimodal feature extraction. The figure shows the feature extraction process of one LncRNA–protein pair. Firstly, sequence features, motif information, physicochemical properties and secondary structure features of the lncRNA and protein are extracted, respectively. The same groups of features of lncRNA and protein are concatenated respectively, yielding four feature vectors. $F_1$–$F_4$ stands for sequence features, motif information, physicochemical properties and secondary structure features, respectively. Note that the dimensions of the four feature vectors are not the same. **b** The architecture of Capsule-LPI. The architecture of Capsule-LPI is divided into two parts, the first part is four different feature-learning subnetworks for four feature vectors, each subnetwork consists of fully connected layers. The second part is one capsule network. $F_1$–$F_4$ are feature vectors, refer to sequence feature, motif information, physicochemical properties and secondary structure, respectively. First, each feature vector passes through its own feature learning subnetwork to get a three-dimensional output vector. Then the output vectors are treated as capsules and obtain $U_1$–$U_4$. $U_1$–$U_4$ include diversified information with prediction by each feature. $W_1$–$W_4$ are transformation matrices. They are able to transform $U_1$–$U_4$ into the same prediction space and they are the only parameters learned through backpropagation in the second part. $U'_1$–$U'_4$ represent the predictions of different features in the same prediction space. Next, add $U'_1$–$U'_4$ to get a new capsule. Using the “squashing” activation function to compress the length of the new capsule to a range from 0 to 1 to get the final Capsule *V*. Then take the length of *V* to represent the final prediction result, with lengths greater than 0.5 as interactions and lengths less than 0.5 as no interactions. Note that $U_1$–$U_4$, $U'_1$–$U'_4$ and *V* are essentially vectors; we call them “capsules” because they are the units of capsule network

The flowchart of Capsule-LPI is shown in Fig. 1. Capsule-LPI includes two steps: (a) Multimodal feature extraction. When Capsule-LPI received a LncRNA–protein pair, four groups of features, including sequence features, motif information, physicochemical properties and secondary structure features, could be extracted automatically. Each group of features, after being extracted, forms a feature vector. Therefore, four different feature vectors are obtained. (b) The architecture of Capsule-LPI. The architecture of Capsule-LPI consists of four feature-learning subnetworks and one capsule subnetwork [35]. The feature-learning subnetwork consists of fully connected layers. Four different feature vectors obtained by multimodal feature extraction are input into the feature learning subnetworks to automatically learn the more informative and high-level features. Then, capsule subnetworks further integrate the features and predict LPI.

### Data description

The dataset is downloaded from NPInter database [38]. The database removes lncRNA–protein interacting pairs for nonhuman species and ncRNAs less than 200 nt in length. A total of 6204 lncRNA–protein interacting pairs were eventually retained. No negative lncRNA–protein samples existed in the database, so we needed to construct negative samples. We use the method of negative sample construction used in existing methods for LPI prediction [15]. The process of generating a negative sample set was as follows: first, all the lncRNAs and proteins used in the positive sample were obtained from the NPInter database, and there were 2356 lncRNAs and 90 proteins in total. Then, the 2356 lncRNAs and 90 proteins were combined one by one, resulting in a total of 212,040 LncRNA–protein pairs. Finally, the 6204 LncRNA–protein pairs in the positive sample were removed, and 205,836 (21,040-6,204) LncRNA–protein pairs were considered negative samples. The imbalance between large positive samples and negative samples can lead to prediction bias [39], so we randomly divided 205,836 negative samples into 33 sets, and each set contained 6204 negative samples. Thus far, we have obtained 6204 lncRNA–protein interacting pairs and 33 sets consisting of 6204 lncRNA–protein noninteracting pairs, which can be obtained at http://csbg-jlu.site/lpc/download. Each lncRNA–protein noninteracting pair set was combined with a lncRNA–protein interacting pair set to train one model. Then, we adopted EasyEnsemble [40] to ensemble 33 models to obtain the final model. More details refer to (Additional file 1: S1).

### Multimodal features extraction

To capture more perspectives of LPI, four types of features (sequence features, motif information, physicochemical properties and secondary structure features) are extracted. The extraction process of multimodal features is shown in Fig. 1a. The following four subsections are detailed descriptions of each type of feature.

#### Sequence features

For lncRNAs, 4-mer frequency features are chosen to encode each lncRNA with a 256 ($4\,\times\, 4\,\times\, 4\,\times\, 4$) dimensional vector, and each element of the vector corresponds to the frequency of the corresponding 4-mer (e.g., AUUC, AACG, CGUC) in the sequence of lncRNAs. The formula for calculating the frequency is as follows:

1 $$\begin{aligned} f_{i} = \frac{n_{i}}{\sum _{j=1}^{256}n_j} \end{aligned}$$

where *i* is a serial number, $f_{i}$ is the k-mer frequency of the *i*-th k-mer and $n_{i}$ represents the number of *i*-th k-mer in the sequence.

For proteins, to reduce the feature dimension, the Novo method [22] is used to classify amino acids into four groups: {D, E}, {H, R, K}, {C, G, N, Q, S, T, Y, A}, {F, I, L, M, P, V, W}. Then 3-mer frequency features are chosen to encode each protein with a 64 (4$\times$4$\times$4) dimensional vector, and each element of the vector corresponds to the frequency of the corresponding 3-mer in the sequence of the protein. The calculation of frequency refers to Formula 1.

We selected 4-mer frequency features and 3-mer frequency features to encode each lncRNA and protein sequence, respectively, because smaller k-values are poor representations of the sequence, while larger k easily results in sparse representation. In the existing models, the 4-mer frequency features for lncRNA and 3-mer frequency features for protein are mostly considered for LPI prediction [12, 41–43].

In total, the dimension of the sequence feature vector of each lncRNA–protein pair was 320 (256+64).

#### Motif information

Many motifs have been found to be helpful to predict RNA-protein interactions [44–46]. We use the number of each motif in the sequence to form motif features. Each lncRNA is encoded with an 18-dimensional vector corresponding to 18 motifs: Fox1, Nova, Slm2, Fusip1, PTB, ARE, hnRNPA1, PUM, U1A, HuD, QKI, U2B, SF1, HuR, YB1, {AU}, {UG} and a motif group, which combines Fox1, Nova, ARE, PUM and U1A. Each protein is encoded with an 11-dimensional vector corresponding to 11 motifs: {H, R}, {HR, RH}, {E}, {K}, {H}, {R}, {EE}, {KK}, {RS, SR}, {RGG} and {YGG}. The details of each motif are provided in (Additional file 1: S2).

In total, the dimension of the motif feature vector of each lncRNA–protein pair was 29 (18 + 11).

#### Physicochemical properties

The physicochemical properties were used to predict LPI in lncPro [13] and LncADeep [25]. In Capsule-LPI, we adopt the physicochemical properties used in lncPro and add some other physicochemical properties. For lncRNAs, van der Waals interactions and hydrogen-bonding propensities [47] were used to encode each lncRNA sequence into 2 numerical vectors. For proteins, Bull & Breese hydrophobicity [48], Kyte & Doolittle hydrophobicity [49], Zimmerman polarity [50], Grantham polarity [51], isoelectric point, bulkiness, Eisenberg hydrophobicity [52] and Hopp & Woods hydrophobicity propensities [53] were used to encode each protein sequence into 8 numerical vectors. These physicochemical properties are selected because they have been validated by many LPI methods [13, 25].

However, because the dimension of each feature vector depends on the length of the corresponding lncRNA or protein sequence, the input feature vector dimensions of different samples are different. Therefore, the vectors need to be transformed to the same dimension. Here, we adopt the method in lncPro, and use the Fourier transform, which is applied to transform two physicochemical properties into a spectrum domain. The formula of the Fourier series is as follows:

2 $$\begin{aligned} X_{k}^{'} = \sqrt{\frac{2}{L}}\sum _{n=0}^{L}X_n\cos \left[ \frac{\pi }{L}\left( n+\frac{1}{2}\right) \left( k+\frac{1}{2}\right) \right] , k=0, 1, ..., 9 \end{aligned}$$

where *L* is the length of the original feature vector and $X_{n}$ is the *n*-th value in the original feature vector. The first 10 terms of the Fourier series were used as a new spectrum feature vector. Each lncRNA sequence was encoded into two 10-dimensional spectrum vectors corresponding to its two physicochemical feature vectors. Each protein sequence was encoded into eight 10-dimensional spectrum vectors corresponding to its 8 physicochemical feature vectors.

In total, the dimension of the physicochemical spectrum property feature vector of each lncRNA–protein pair was 100 (2 $\times$ 10 + 8 $\times$ 10).

#### Secondary structure features

The secondary structure of lncRNAs and proteins is more conserved than the sequence, which is an important feature to infer LPI. The secondary structure of each lncRNA was obtained using RNAfold [54] based on the minimum free energy algorithm. Then, we transferred the secondary structure to a numerical vector consisting of 0 and 1, in which the paired nucleotide was replaced by 1 and the unpaired nucleotide was replaced by 0.

There is also the problem that the length of the numerical feature vector is related to the length of the sequence, which causes the input vector dimension of different samples to be different. The Fourier transform is used to transform the feature vector and keep the first 10 terms as a new spectrum feature vector. The Fourier series is shown in Formula 2. In this way, the secondary structure feature vector of each lncRNA with dimensions of 10 was obtained.

For the protein secondary structure, the secondary structure sequence of each protein was first obtained using Predator [55]. Then, the secondary structure sequence of each protein was encoded into a numerical feature vector by the Chou-Fasman propensities [56], which were used in the lncPro and LncADeep methods. Each feature vector is also transformed by Fourier transform, and the first 10 terms are retained as a new spectrum feature vector. In this way, the secondary structure feature vector of each protein with dimensions of 10 was obtained.

In total, the dimension of the secondary structure feature vector of each lncRNA–protein pair was 20 (10+10).

Here, the feature vector encoding process was completed. For each lncRNA–protein pair, we obtained 4 groups of feature vectors: sequence feature vector (320-dimensional vector), motif feature vector (29-dimensional vector), physicochemical properties feature vector (100-dimensional vector) and secondary structure feature vector (20-dimensional vector).

### Architecture of capsule-LPI

The key architecture of Capsule-LPI is divided into two parts, as shown in Fig. 1b. The first part is four feature-learning subnetworks, and the second part is one capsule subnetwork [35] for prediction. In this section, the architecture of Capsule-LPI and the hyperparameter setting are introduced in detail.

Each feature vector needs one feature-learning subnetwork. Each feature learning subnetwork is made up of fully connected layers, and this subnetwork can not only extract high-level features but also unify the dimensions of feature vectors. The hyperparameters of the feature learning subnetworks are shown in Additional file 1: S3. By experiments, 5 fully connected layers for each subnet are selected because the prediction accuracy does not grow significantly when the layer number is larger than 5, and a larger hidden layer number brings more computation. The number of neurons in each hidden layer was obtained through multiple experiments. PReLU is used as an activation function. To prevent overfitting, we add dropout layers [57] to the hidden layers.

Then, each feature vector is fed into its own feature learning subnet, and the output of each vector is obtained with dimension 3. The dimension 3 was chosen because after trying multiple output dimensions, when the output dimension of the feature extraction subnets is 3, the prediction accuracy of the model is the highest.

The second part of the architecture is a capsule network. The novel learned high-level abstract feature vectors from feature learning subnets are treated as capsules and further fed into the capsule subnetwork. A capsule is essentially a vector; we call it a “capsule” because it is the unit of the capsule network and needs to be distinguished from the vector. A capsule is a group of neurons, and as opposed to a single neuron, a capsule contains more information [35]. As shown in Fig. 1b, $U_1$–$U_4$ are capsules corresponding to four high-level abstract features, which contain multiple predicted information on LPI. $W_1$–$W_4$ are transformation matrices that are able to transform $U_1$–$U_4$ into the same prediction space and they are the only parameters learned through backpropagation in the second part. $U'_1$–$U'_4$ are the predictions of different features in the same prediction space. The length of $U'_i$ represents the interaction rate of LncRNA–protein obtained by prediction with the $i-th$ feature, and its direction represents other information on LPI. If $U'_1$–$U'_4$ are long in length and close in orientation, these properties indicate that the multiple features support LPI in terms of prediction propensity, as well as other interaction information stored in capsules; if $U'_1$–$U'_4$ are long but differ in orientation, these properties indicate that only the prediction propensity of each feature supports LPI, but other interaction information stored in capsules is not sufficient to support LPI. This network makes the prediction, considering not only the predictive tendencies of each feature but also other interaction information and the relationships between the different features.

To determine whether the capsules ($U'_1$–$U'_4$) mostly agree with LPI in terms of prediction propensity (reflected in the length of the capsules) as well as other interaction information (reflected in the orientation of the capsules), we add these capsules to obtain a new capsule, *S*. If S is long, the length of S shows that most of the capsules ($U'_1$–$U'_4$) are long and the capsules are oriented similarly, indicating that $U'_1$–$U'_4$ mostly agree with LPI and their other stored information also fits. We do not use the dynamic routing algorithm that is used in the capsule network paper in the adding step because we are a biclassing problem that only needs to output one capsule, which does not require a dynamic routing algorithm. The architecture of the capsule network is shown in (Additional file 1: S3).

Use the length of the final output capsule to represent the LPI’s possibility. Therefore, the “squashing” activation function [35] in the capsule network is used to ensure that the short vector shrinks to almost 0 length and the long vector shrinks to a length slightly below 1. The “squashing” activation function formula is:

3 $$\begin{aligned} V = \frac{||S||^{2}}{1+||S||^{2}}\frac{S}{||S||} \end{aligned}$$

where *V* is the output capsule, and *S* is the sum of $U'_1$–$U'_4$.

Finally, take the length of *V* to represent the predictions, with lengths greater than 0.5 as interactions and lengths less than 0.5 as no interactions.

### Evaluation criteria

To evaluate the performance of Capsule-LPI, we use six evaluation metrics: AUC, AUPRC, accuracy, precision, recall, and F-value. AUC and AUPRC are the area under the ROC and P-R curves, respectively. The formulas for the rest of the evaluation metrics are as follows:

4 $$\begin{aligned} Accuracy= & {} \frac{TP+TN}{TP+TN+FP+FN} \end{aligned}$$

5 $$\begin{aligned} Precision= & {} \frac{TP}{TP+FP}\end{aligned}$$

6 $$\begin{aligned} Recall= & {} \frac{TP}{TP+FN}\end{aligned}$$

7 $$\begin{aligned} F-value= & {} \frac{2\times Precision\times Recall}{Precision+Recall} \end{aligned}$$

where *TP*, *FP*, *TN* and *FN* represent true positives, false positives, true negatives and false negatives, respectively. *TP* is the number of samples in the test set for which the prediction result is positive and the label is also positive. *FP* is the number of samples in the test set for which the prediction result is positive but the label is negative. *TN* is the number of samples in the test set for which the prediction result is negative and the label is also negative. And *FN* is the number of samples in the test set for which the prediction result is negative but the label is positive. *Precision* reflects the confidence level when the outcome prediction is positive. *Sensitivity* reflects the probability that we capture the sample when the sample is positive. *Accuracy* and $F-value$ are composite measures used to evaluate the comprehensive performance score.

## Results

Three experiments have been conducted to evaluate Capsule-LPI in terms of architecture, feature combination, and overall performance.

### Architecture comparison

First, it is necessary to evaluate the performance of the architecture of Capsule-LPI. Since Capsule-LPI uses a deep learning architecture, we built three deep learning frameworks, fully connected network (FC), CNN and LSTM, to compare with the architecture of Capsule-LPI. In addition, we also compared the architecture of the existing LPI tool such as the deep stacking network architecture of LncADeep [25]. The architectures were tested on four kinds of features designed in our work (sequence features, motif information, physicochemical properties and secondary structure features). Moreover, a set of control experiments between the architecture of Capsule-LPI and LncADeep using the features of LncADeep was also added to fully assess the performance of the architecture of Capsule-LPI. The features of LncADeep include sequence features and structural features (Additional file 1: S4). The performances of the architecture of Capsule-LPI and other architectures with 10-fold cross-validation are shown in Table 1.

**Table 1:** Comparison of the performances of the Capsule-LPI with other deep learning architectures under 10-fold cross-validation

Tools	AUC (%)	AUPRC (%)	Accuracy (%)	Precision (%)	Recall (%)	F-value (%)
FC	$95.19\pm 0.73$	$92.93\pm 1.18$	$90.96\pm 0.86$	$\mathbf{88}.75 \pm \mathbf{1}.61$	$93.87\pm 0.77$	$91.22\pm 0.76$
CNN	$94.64\pm 0.78$	$92.25\pm 1.30$	$89.70\pm 1.22$	$87.24\pm 1.51$	$93.03\pm 1.11$	$90.03\pm 1.14$
LSTM	$93.26\pm 0.84$	$88.66\pm 1.64$	$89.70\pm 0.89$	$88.13\pm 1.37$	$91.82\pm 1.94$	$89.91\pm 0.91$
LncADeep	$90.55\pm 1.18$	$85.33\pm 2.04$	$87.73\pm 1.03$	$83.89\pm 1.45$	$93.45\pm 1.64$	$88.39\pm 0.96$
Capsule-LPI	$\mathbf{95}.31 \pm \mathbf{0}.41$	$\mathbf{93}.30 \pm \mathbf{0}.92$	$\mathbf{91}.66 \pm \mathbf{0}.86$	$88.15\pm 0.86$	$96.25\pm 0.90$	$\mathbf{92}.02 \pm \mathbf{0}.82$
LncADeep (Use LncADeep’s features)	$89.52\pm 0.84$	$84.16\pm 1.60$	$87.29\pm 1.04$	$83.70\pm 1.69$	$92.69\pm 1.81$	$87.94\pm 0.97$
Capsule-LPI (Use LncADeep’s features)	$95.11\pm 0.50$	$93.19\pm 0.65$	$91.34\pm 1.01$	$87.11\pm 1.48$	$\mathbf{97}.08 \pm \mathbf{0}.85$	$91.82\pm 0.91$

Table 1 shows that under the same features as well as the same test environment, the architecture of Capsule-LPI achieved better performance than the architecture of other deep-learning architectures. On AUC, AUPRC, accuracy, recall and F-value, Capsule-LPI achieves 95.31%, 93.30%, 91.66%, 96.25% and 92.02% under 4 features, respectively, which are all higher than FC, CNN, LSTM and deep stacking network architecture. The architecture of Capsule-LPI has the greatest improvement in the recall metric, which increases close to 3%. The high recall index means that the architecture of Capsule-LPI can identify more potential LPIs. The F-value has a nearly 1% increase, indicating that the overall performance of Capsule-LPI is better. To further evaluate whether the improvement of the Capsule-LPI architecture is significant, we calculated the p-values of the F-value between the Capsule-LPI architecture and other deep learning architectures using the paired t-test on the results of ten iterations. The p-values of F-value for different architecture comparisons are listed as follows: 6.53e-3 for Capsule-LPI vs FC, 1.58e-4 for Capsule-LPI vs CNN, 1.56e-5 for Capsule-LPI vs LSTM, 4.53e-7 for Capsule-LPI vs lncADeep, 8.13e-7 for Capsule-LPI (Use features of LncADeep) vs lncADeep (Use features of LncADeep). All p-values are less than 0.05, which shows that the improvement is significant. The architecture of the performance of Capsule-LPI is also higher than the performance of LncADeep when using the features of LncADeep, indicating that the architecture of Capsule-LPI is not only dependent on the 4 features mentioned in this paper.

### Evaluation of combinations of different features

After verifying that the architecture of Capsule-LPI performs well, the multimodal features that are appropriate for Capsule-LPI need to be selected. Here, four features are evaluated. To understand whether each feature is valid in predicting and what combination of features is the best choice, we conducted 15 experiments to evaluate the performance of different features and feature combinations using the Capsule-LPI architecture. The architecture needs to be fine-tuned when inputting different feature combinations. When some features are not adopted, the Capsule-LPI architecture only needs to close the corresponding channel of these features. The architectures of Capsule-LPI for different numbers of feature combinations are shown in Additional file 1: S5. The results for different combinations under 10-fold cross-validation are shown in Table 2.

**Table 2:** Comparison of the performance of different features and different feature combinations under 10-fold cross-validation

Feature Combinations	AUC (%)	AUPRC (%)	Accuracy (%)	Sensitivity (%)	Precision (%)	F-value (%)
SF (Sequence feature)	$94.16\pm 0.79$	$89.99\pm 1.66$	$90.78\pm 0.74$	$87.48\pm 1.20$	$95.20\pm 0.59$	$91.17\pm 0.65$
Mtf (Motif information)	$87.97\pm 2.37$	$82.01\pm 4.70$	$85.20\pm 1.28$	$78.99\pm 1.67$	$96.00\pm 0.73$	$86.66\pm 1.04$
PC (Physicochemical)	$93.89\pm 0.72$	$90.66\pm 1.47$	$88.99\pm 1.07$	$83.15\pm 1.28$	$\mathbf{97}.83 \pm \mathbf{0}.97$	$89.89\pm 0.93$
SS (Secondary structure)	$89.46\pm 2.38$	$85.35\pm 4.98$	$83.68\pm 1.60$	$78.59\pm 1.79$	$92.67\pm 2.32$	$85.03\pm 1.42$
SF + Mtf	$94.43\pm 0.89$	$91.37\pm 1.43$	$91.03\pm 1.03$	$87.33\pm 1.64$	$96.03\pm 0.85$	$91.46\pm 0.91$
SF + PC	$95.33\pm 0.63$	$93.42\pm 0.93$	$91.49\pm 1.02$	$87.76\pm 1.71$	$96.48\pm 0.93$	$91.90\pm 0.90$
SF + SS	$94.79\pm 0.77$	$92.31\pm 1.41$	$90.83\pm 0.99$	$86.95\pm 1.27$	$96.09\pm 1.00$	$91.29\pm 0.91$
Mtf + PC	$94.01\pm 0.80$	$90.82\pm 1.34$	$89.00\pm 1.12$	$83.24\pm 1.60$	$97.74\pm 1.35$	$89.89\pm 0.97$
Mtf + SS	$91.37\pm 1.30$	$89.45\pm 1.83$	$84.79\pm 1.10$	$77.96\pm 1.44$	$97.08\pm 0.74$	$86.46\pm 0.83$
PC + SS	$94.12\pm 0.61$	$91.66\pm 0.60$	$89.12\pm 1.02$	$83.53\pm 1.19$	$97.50\pm 1.12$	$89.97\pm 0.91$
Mtf + PC + SS	$94.13\pm 0.81$	$91.14\pm 1.55$	$89.02\pm 1.18$	$84.14\pm 1.70$	$96.24\pm 2.12$	$89.76\pm 1.09$
SF + PC + SS	$95.23\pm 0.65$	$93.17\pm 0.96$	$91.41\pm 1.02$	$86.94\pm 1.43$	$97.48\pm 0.71$	$91.90\pm 0.91$
SF + Mtf + SS	$94.73\pm 0.57$	$92.21\pm 0.86$	$90.75\pm 0.82$	$86.98\pm 1.44$	$95.90\pm 0.87$	$91.21\pm 0.71$
SF + Mtf + PC	$\mathbf{95}.42 \pm \mathbf{0}.51$	$93.27\pm 1.00$	$91.62\pm 0.80$	$\mathbf{88}.46 \pm \mathbf{1}.46$	$95.79\pm 0.98$	$91.96\pm 0.71$
SF + Mtf + PC + SS	$95.31\pm 0.41$	$\mathbf{93}.30 \pm \mathbf{0}.92$	$\mathbf{91}.66 \pm \mathbf{0}.86$	$88.15\pm 0.86$	$96.25\pm 0.90$	$\mathbf{92}.02 \pm \mathbf{0}.82$

As shown in Table 2, the single feature of physicochemical properties had the highest recall score, which was 97.83%. The combination of sequence features, motif information, and physicochemical properties yielded the highest AUC score and precision score, which were 95.42% and 88.46%, respectively. For the AUPRC, accuracy and F-value, the combination of 4 features obtained the highest values of 93.30%, 91.66% and 92.02%, respectively. Among the six evaluation indexes, three comprehensive indexes of the combination of 4 features obtained the highest scores, so the combination of 4 features can be considered to be more suitable for the architecture of Capsule-LPI.

### Comparison of capsule-LPI performance with existing tools

After verifying the architecture of Capsule-LPI and selecting the suitable feature combinations for Capsule-LPI, the overall performance of Capsule-LPI needs to be evaluated. Several state-of-the-art tools for predicting RNA-protein interactions are compared, i.e., RPISeq [12], lncPro [13], RPI-pred [14], rpiCool [15], IPMiner [24] and LncADeep [25]. For the experiments to be comparable, Capsule-LPI is evaluated concerning the methodology in LncADeep, in which the same dataset used in lncADeep, as well as the evaluation method, are adopted. For the same data set, Capsule-LPI uses the exact same positive sample as LncADeep. Since LncADeep does not provide the negative sample, Capsule-LPI uses the same negative sample generation method as LncADeep. For the same evaluation method, 5-fold cross-validation, which is used in LncADeep, is used to evaluate the performance of Capsule-LPI. The comparison results are shown in Table 3.

**Table 3:** Comparison of performances for predicting lncRNA–protein interaction by Capsule-LPI and other tools under 5-fold cross validation

Tools	Sensitivity (%)	Precision (%)	F-value (%)
RPISeq(RF)	$\mathbf{99}.1 \pm \mathbf{0}.2$	$50.1\pm 0.1$	$66.5\pm 0.1$
RPISeq(SVM)	$93.5\pm 0.7$	$50.2\pm 0.2$	$65.3\pm 0.4$
lncPro	$80.3\pm 0.9$	$52.2\pm 0.6$	$63.2\pm 0.4$
RPI-pred	$88.0\pm 0.3$	$49.8\pm 0.6$	$63.6\pm 0.5$
rpiCool	$92.0\pm 0.8$	$83.3\pm 0.8$	$87.5\pm 0.6$
IPMiner	$89.8\pm 1.1$	$85.6\pm 0.7$	$87.6\pm 0.6$
LncADeep	$97.0\pm 0.5$	$85.4\pm 0.8$	$90.8\pm 0.4$
Capsule-LPI	$97.6\pm 0.6$	$\mathbf{87}.3 \pm \mathbf{0}.2$	$\mathbf{92}.2 \pm \mathbf{0}.3$

As shown in Table 3, under the 5-fold cross validation averaging assessment condition, Capsule-LPI with 87.3% precision and 92.2% F-value is superior to other existing tools. Since other tools do not support retraining, we only calculated the AUC and AUPRC of Capsule-LPI under 5-fold cross validation, which were $95.28\pm 0.47\%$ and $95.26\pm 0.62\%$, respectively. The precision of Capsule-LPI was at least 1.7% higher than the precision of the other tools, indicating that the LncRNA–protein interaction pairs obtained by Capsule-LPI prediction are highly reliable. The F-value of Capsule-LPI also achieves a 1.4% improvement, showing that the overall performance of Capsule-LPI is the best. For sensitivity, Capsule-LPI obtains 97.6%, which is slightly lower than the highest sensitivity of 99.1% obtained by RPISeq (RF). However, RPISeq does not perform well on the other two metrics, and its precision is only 50.1%. Therefore, overall Capsule-LPI outperforms the outstanding current tools.

## Discussion

In the results section, three experiments have evaluated the performance of Capsule-LPI. First, to verify the architecture of Capsule-LPI, it was tested against four architectures. The experimental results show that the architecture of Capsule-LPI outperforms these four architectures, which shows that it is an effective architecture. Second, a comprehensive feature evaluation experiment is conducted. We consider four kinds of features used in the existing LPI prediction tools and select the best combination of features for Capsule-LPI, which contains sequence features, motif information, physicochemical properties and secondary structure features. Finally, Capsule-LPI is compared to other outstanding LPI prediction tools. The results show that Capsule-LPI outperforms state-of-the-art methods in LPI prediction.

However, a good tool should not only have good performance but also be helpful to scientific research and easy to use. In this regard, we have done a case study to introduce how this work can help with the research of lncRNAs and develop a webserver that is convenient to use.

### One case study: finding lncRNA related diseases

**Fig. 2:**
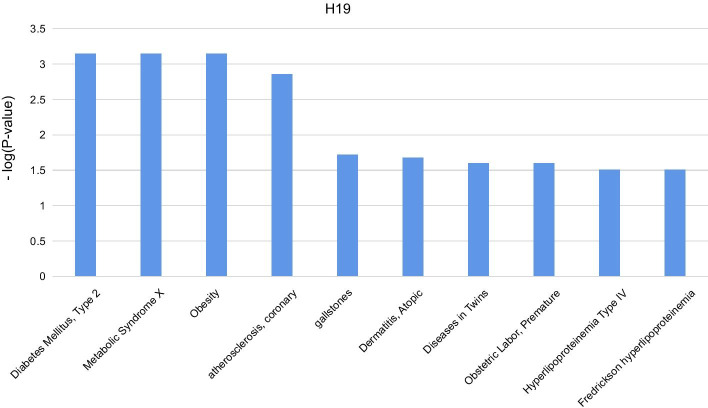
The potential disease association results of H19

To demonstrate the effectiveness and practicability of Capsule-LPI for follow-up research on lncRNAs, a case study for lncRNA-disease association was conducted. In this case, study, we used Capsule-LPI to predict which proteins interact with the top 10 lncRNAs of interest on PubMed and less studied lncRNAs on PubMed. Then, for each lncRNA, the diseases were inferred according to the enriched diseases of the predicted interacting proteins computed by hypergeometric distribution inference. The process of the case study is as follows:

1. We queried the PubMed database to obtain H19, MALAT1, HOTAIR, MEG3, NEAT1, GAS5, UCA1, XIST, PVT1 and TUG1 and downloaded the sequences of these lncRNAs.
2. A total of 26,560 protein sequences of Homo sapiens were downloaded from the UniProt database.
3. Capsule-LPI was used to predict the interacting proteins for lncRNAs, in which the threshold value was set to 0.87 for the higher confidence of the LPI results.
4. The potential disease association of each lncRNA was inferred by the enrichment analysis of disease association for the interacting proteins through the DAVID online service website. Here, we show the potential disease association results of H19 in Fig. 2, and the rest of the lncRNAs are referred to (Additional file 1: S6).

Furthermore, potentially related diseases are categorized into different types of diseases, including metabolic, chemical dependence, cardiovascular, immune, pharmacogenomic, cancer, infection, neurological, renal, ageing, developmental, haematological, psych, reproduction and vision. The top 10 lncRNAs on PubMed are highly related to metabolic, chemical dependence, cardiovascular, immune and pharmacogenomic processes. To test the validity of this result, we searched the LncRNADisease database for the diseases corresponding to each lncRNA, and the vast majority of the diseases were covered by our predictions. For example, the diseases currently known to be associated with H19 are coronary artery disease, gastric cancer, neural tube defects, kidney cancer, infertility, etc., which correspond to cardiovascular, cancer, neurological, renal, and reproduction, respectively.

**Fig. 3:**
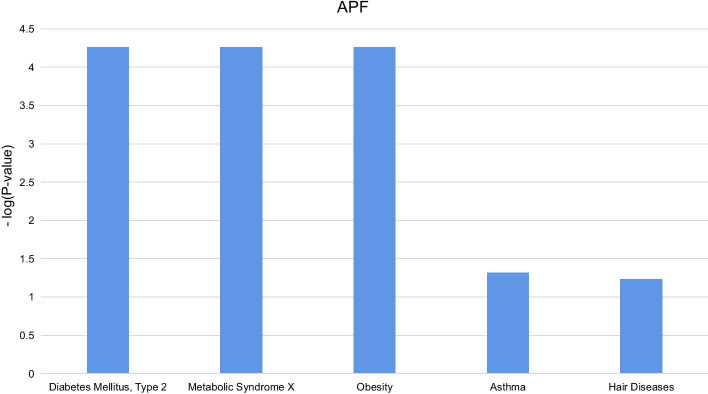
The potential disease association results of APF

5. For some less studied lncRNAs on PubMed and LncRNADisease, the same process is executed. The potential disease association results of APF are shown in Fig. 3. Here, APF lncRNA was selected with only 2 reports in PubMed and only associated with myocardial infarction disease in the LncRNADisease database. By conducting the above process, more disease associations are inferred, including diabetes mellitus type 2, metabolic syndrome X, obesity, asthma and hair diseases. These unreported diseases can provide insightful directions for future work with biological and medical researchers.

All the sequences of lncRNAs, proteins, predicted interacting proteins and inferred potential disease associations for each lncRNA can be downloaded at our website (http://csbg-jlu.site/lpc/download). This case study demonstrates the effectiveness and practicability of our Capsule-LPI tool. Furthermore, with the interacting proteins predicted by Capsule-LPI, we can not only analyse their association with disease but also gain more information inference for lncRNAs such as function, evolution and subcellular localization.

### Webserver of capsule-LPI

**Fig. 4:**
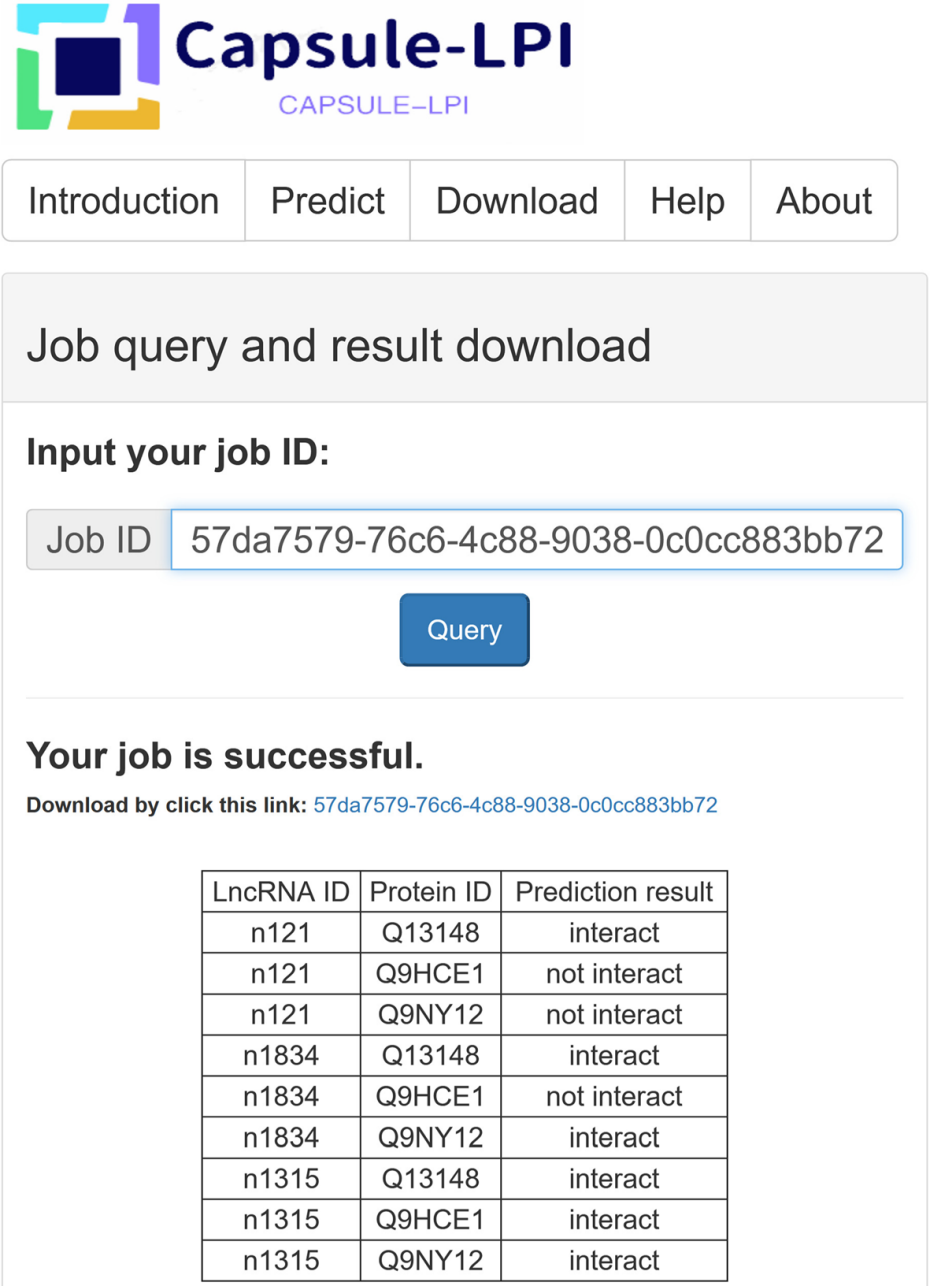
The screenshot of Capsule-LPI’s webserver

We developed a webserver of Capsule-LPI with a user-friendly interface to facilitate users. The screenshot of the webserver of Capsule-LPI is shown in Fig. 4. Capsule-LPI allows online prediction of large volumes of data. The upper input limit for both lncRNA and protein sequences is 100, which means 10000 LncRNA–protein pairs can be predicted online. For each task submitted, a job ID can be assigned, and the prediction results can be downloaded by this job ID. For more functions and help, please refer to “help” on the website.

## Conclusions

Identifying LPI is key to understanding lncRNA functions. Compared to experimental methods, computational methods are much more economical and efficient. Although some LPI prediction computational methods have been developed, how to integrate multimodal features from more perspectives and build deep learning architectures with better recognition performance has always been a challenging research highlight. Inspired by the better theory and the improved performance of the capsule network than CNN acting in image recognition, we propose a novel multichannel capsule network framework (Capsule-LPI) to integrate multimodal features for LPI prediction.

Capsule-LPI integrates four groups of multimodal features, including sequence features, motif information, physicochemical properties and secondary structure features. The architecture of Capsule-LPI is composed of four feature learning subnetworks and one capsule subnetwork. The multichannel framework of Capsule-LPI can make it better to integrate and learn multiple features by considering not only the predictive tendencies of each feature but also other interaction information and the relationships between different features.

To comprehensively evaluate the performance of Capsule-LPI, three different kinds of experiments were conducted: (i) tool architecture comparison; (ii) evaluation of different feature combinations; and (iii) comparison with existing tools. Through comprehensive experimental comparisons and evaluations, we demonstrated that both multimodal features and the architecture of a multichannel capsule network can significantly improve the performance of LPI prediction. Capsule-LPI performs better than existing state-of-the-art tools. A webserver (http://csbg-jlu.site/lpc/predict) has been developed to be convenient for users.

This study provides a novel and feasible LPI prediction tool based on the integration of multimodal features and a capsule network. In the future, we expect to integrate more advanced structural features and external association information to further improve the accuracy of Capsule-LPI and provide more convenient and practical tools for researchers.

## Supplementary information

**Additional file 1.** Supplementary materials for Capsule-LPI Contains: S1. Dataset for Capsule-LPI; S2. The details of motifs; S3. The architecture of Capsule-LPI; S4. Features of LncADeep used in architecture comparison section; S5. Samples of the architectures of Capsule-LPI for different number of feature combination; S6. The potential lncRNA-disease association results.

## Data Availability

The source code of Capsule-LPI and datasets can be accessed at the following URL: http://csbg-jlu.site/lpc/download/.

## Abbreviations

lncRNAs: Long noncoding RNAs
LPIs: LncRNA–protein interactions
LPI: LncRNA–protein interaction
SVM: Support vector machine
RF: Random forests
DNN: Deep neural network
FC: Fully connected network

## References

[1] Gutschner T, Diederichs S (2012). The hallmarks of cancer: A long non-coding rna point of view. RNA Biology.

[2] Guttman M, Rinn JL (2012). Modular regulatory principles of large non-coding rnas. Nature.

[3] Pang KC, Frith MC, Mattick JS (2006). Rapid evolution of noncoding rnas: lack of conservation does not mean lack of function. Trends Genet.

[4] Kutter C, Watt S, Stefflova K, Wilson MD, Goncalves A, Ponting CP, Odom DT, Marques AC (2012). Rapid turnover of long noncoding rnas and the evolution of gene expression. PLoS Genet.

[5] Kung JT, Colognori D, Lee JT (2013). Long noncoding rnas: past, present, and future. Genetics.

[6] Wilusz JE, Sunwoo H, Spector DL (2009). Long noncoding rnas: functional surprises from the rna world. Genes Dev.

[7] Harries LW (2012). Long non-coding rnas and human disease. Biochem Soc Trans.

[8] Fu M, Zou C, Pan L, Liang W, Qian H, Xu W, Jiang P, Zhang X (2016). Long noncoding rnas in digestive system cancers: Functional roles, molecular mechanisms, and clinical implications (review). Oncol Rep.

[9] Rathinasamy B, Velmurugan BK (2018). Role of lncrnas in the cancer development and progression and their regulation by various phytochemicals. Biomedicine & Pharmacotherapy.

[10] Dangelmaier, E., Lal, A.: Adaptor proteins in long noncoding rna biology. Biochimica et Biophysica Acta (BBA)–Gene Regulatory Mechanisms 1863, 194370 (2020) 10.1016/j.bbagrm.2019.03.003 30951902

[11] McHugh, C., Russell, P., Guttman, M.: Mchugh, ca, russell, p and guttman, m. methods for comprehensive experimental identification of rna-protein interactions. genome biol 15: 203. Genome biology 15, 203 (2014) 10.1186/gb4152 PMC4054858 24467948

[12] Muppirala UK, Honavar VG, Dobbs DJBB (2011). Predicting rna-protein interactions using only sequence information.

[13] Lu Q, Ren S, Lu M, Zhang Y, Zhu D, Zhang X, Li T (2013). Computational prediction of associations between long non-coding rnas and proteins. BMC Genomics.

[14] Suresh V, Liu L, Adjeroh D, Zhou X (2015). Rpi-pred: predicting ncrna-protein interaction using sequence and structural information. Nucleic Acids Research.

[15] Akbaripour-Elahabad, M., Zahiri, J., Rafeh, R., Eslami, M., Azari, M.J.J.o.T.B.: rpicool: A tool for in silico rna–protein interaction detection using random forest **402**, 1–8 (2016) 10.1016/j.jtbi.2016.04.025 27134008

[16] Li A, Ge M, Zhang Y, Peng C, Wang M (2015). Predicting long noncoding rna and protein interactions using heterogeneous network model. Biomed Res Int.

[17] Ge M, Li A, Wang M (2016). A bipartite network-based method for prediction of long non-coding rna-protein interactions. Genomics Proteomics Bioinformatics.

[18] Zhang W, Qu Q, Zhang Y, Wang W (2018). The linear neighborhood propagation method for predicting long non-coding rna-protein interactions. Neurocomputing.

[19] Zhang W, Yue X, Tang G, Wu W, Huang F, Zhang X (2018). Sfpel-lpi: Sequence-based feature projection ensemble learning for predicting LncRNA–protein interactions. PLoS Comput Biol.

[20] Zhao Q, Yu H, Ming Z, Hu H, Ren G, Liu H (2018). The bipartite network projection-recommended algorithm for predicting long non-coding rna-protein interactions. Mol Ther Nucleic Acids.

[21] Zhao Q, Zhang Y, Hu H, Ren G, Zhang W, Liu H (2018). Irwnrlpi: Integrating random walk and neighborhood regularized logistic matrix factorization for LncRNA–protein interaction prediction. Front Genet.

[22] Hu H, Zhang L, Ai H, Zhang H, Fan Y, Zhao Q, Liu H (2018). Hlpi-ensemble: Prediction of human LncRNA–protein interactions based on ensemble strategy. RNA Biol.

[23] Yi HC, You ZH, Cheng L, Zhou X, Jiang TH, Li X, Wang YB (2020). Learning distributed representations of rna and protein sequences and its application for predicting LncRNA–protein interactions. Comput Struct Biotechnol J.

[24] Pan X, Fan YX, Yan J, Shen HB (2016). Ipminer: hidden ncrna-protein interaction sequential pattern mining with stacked autoencoder for accurate computational prediction. BMC Genomics.

[25] Cheng, Y., Yang, L., Man, Z., Xie, H., Zhang, C., Wang, M.D., Zhu, H.J.B.: Lncadeep: An ab initio lncrna identification and functional annotation tool based on deep learning, 22 (2018) 10.1093/bioinformatics/bty428 29850816

[26] Zhang SW, Zhang XX, Fan XN, Li WN (2020). Lpi-cnncp: Prediction of LncRNA–protein interactions by using convolutional neural network with the copy-padding trick. Anal Biochem.

[27] Zhang Y, Jia C, Kwoh CK. Predicting the interaction biomolecule types for lncrna: an ensemble deep learning approach. Brief Bioinform. 2020. 10.1093/bib/bbaa228 33003205

[28] Wekesa JS, Meng J, Luan Y (2020). A deep learning model for plant LncRNA–protein interaction prediction with graph attention. Mol Genet Genomics.

[29] Wekesa JS, Meng J, Luan Y (2020). Multi-feature fusion for deep learning to predict plant LncRNA–protein interaction. Genomics.

[30] Zeiler, M.D., Fergus, R.: Visualizing and understanding convolutional networks. In: European Conference on Computer Vision

[31] Hinton GEJS (2009). Deep belief networks.

[32] Williams R, Zipser DJNC (2014). A learning algorithm for continually running fully recurrent neural networks.

[33] Schuster, M., Paliwal, K.K.J.I.T.o.S.P.: Bidirectional recurrent neural networks **45**, 2673–2681 (2002)

[34] Laar, P.v.d., Heskes, T., Gielen, S.J.N.N.: Task-dependent learning of attention **10**, 981–992 (1997) 10.1016/s0893-6080(97)00031-2 12662494

[35] Sabour, S., Frosst, N., Hinton, G.E.: Dynamic routing between capsules (2017)

[36] Zhou, J., Cui, G., Zhang, Z., Yang, C., Liu, Z., Wang, L., Li, C., Sun, M.: Graph neural networks: A review of methods and applications (2018)

[37] Deng Y, Xu X, Qiu Y, Xia J, Zhang W, Liu S (2020). A multimodal deep learning framework for predicting drug-drug interaction events. Bioinformatics.

[38] Yuan, J., Wu, W., Xie, C., Zhao, G., Zhao, Y., Chen, R.: Npinter v2.0: an updated database of ncrna interactions. Nucleic Acids Research 42, 104–108 (2013) 10.1093/nar/gkt1057 PMC3965026 24217916

[39] He H, Garcia EA (2009). Learning from imbalanced data. IEEE Transactions on Knowledge and Data Engineering.

[40] Liu XY, Wu J, Zhou ZH. Exploratory undersampling for class-imbalance learning. 2009;39. 10.1109/TSMCB.2008.2007853 19095540

[41] Yi H-C, You Z-H, Huang D-S, Li X, Jiang T-H, Li L-P (2018). A deep learning framework for robust and accurate prediction of ncrna-protein interactions using evolutionary information. Molecular Therapy - Nucleic Acids.

[42] Pan JF, Wang T, Yu YH, Zhang DB (2016). Preparation and thermal properties of non-equilibrium al/ptfe reactive materials. Hanneng Cailiao/Chinese Journal of Energetic Materials.

[43] Shen J, Zhang J, Luo X, Zhu W, Yu K, Chen K, Li Y, Jiang H (2007). Predicting protein-protein interactions based only on sequences information. Proc Natl Acad Sci U S A.

[44] Jiang, P., Singh, M., Coller, H.A., Zavolan, M.J.P.C.B.: Computational assessment of the cooperativity between rna binding proteins and micrornas in transcript decay **9**, 1003075 (2013) 10.1371/journal.pcbi.1003075 PMC3667768 23737738

[45] Pancaldi V, Bähler J. In silico characterization and prediction of global protein-mrna interactions in yeast. NUCLEIC ACIDS RES. 2011;39. 10.1093/nar/gkr160 PMC3152324 21459850

[46] Ray D, Kazan H, Cook KB, Weirauch MT, Najafabadi HS, Li X, Gueroussov S, Albu M, Zheng H, Yang A, Na H, Irimia M, Matzat LH, Dale RK, Smith SA, Yarosh CA, Kelly SM, Nabet B, Mecenas D, Li W, Laishram RS, Qiao M, Lipshitz HD, Piano F, Corbett AH, Carstens RP, Frey BJ, Anderson RA, Lynch KW, Penalva LOF, Lei EP, Fraser AG, Blencowe BJ, Morris QD, Hughes TR (2013). A compendium of rna-binding motifs for decoding gene regulation. Nature.

[47] Morozova N, Allers J, Myers J, Shamoo YJB (2006). Protein-rna interactions: exploring binding patterns with a three-dimensional superposition analysis of high resolution structures.

[48] Bull HB, Breese K (1974). Surface tension of amino acid solutions: A hydrophobicity scale of the amino acid residues. Archives of Biochemistry and Biophysics.

[49] Kyte, J., Doolittle, R.F.J.J.o.M.B.: A simple method for displaying the hydropathic character of a protein **157**, 105–132 (1982) 10.1016/0022-2836(82)90515-0 7108955

[50] Zimmerman, J.M., Eliezer, N., Simha, R.J.J.o.T.B.: The characterization of amino acid sequences in proteins by statistical methods **21**, 170–201 (1968) 10.1016/0022-5193(68)90069-6 5700434

[51] Grantham R (1974). Amino acid difference formula to help explain protein evolution. Science.

[52] Eisenberg, D., Schwarz, E., Komaromy, M., Wall, R.J.J.o.M.B.: Analysis of membrane and surface protein sequences with the hydrophobic moment plot **179**, 125–142 (1984) 10.1016/0022-2836(84)90309-7 6502707

[53] Hopp TP, Woods KR (1981). Prediction of protein antigenic determinants from amino acid sequences.

[54] Lorenz, R., Bernhart, S.H., Höner zu Siederdissen, C., Tafer, H., Flamm, C., Stadler, P.F., Hofacker, I.L.: Viennarna package 2.0. Algorithms for Molecular Biology 6, 26 (2011) 10.1186/1748-7188-6-26 PMC3319429 22115189

[55] Frishman D, Argos P (1996). Incorporation of non-local interactions in protein secondary structure prediction from the amino acid sequence. Protein Engineering, Design and Selection.

[56] Chou, P.Y., Fasman, G.D.J.A.i.E., Biology, R.A.o.M.: Prediction of the secondary structure of proteins from their amino acid sequence **47**, 145–148 (1978) 10.1002/9780470122921.ch2 364941

[57] Srivastava N, Hinton G, Krizhevsky A, Sutskever I, Salakhutdinov R (2014). Dropout: A simple way to prevent neural networks from overfitting. Journal of Machine Learning Research.

