# Supplementary material for: Capsule-LPI: a LncRNA–protein interaction predicting tool based on a capsule network
Source: BMC Bioinformatics. 2021 May 13;22:246. doi: 10.1186/s12859-021-04171-y (PMC8120853; doi:10.1186/s12859-021-04171-y)
Supplement: Supplementary file 1 — Additional file 1. Supplementary materials for Capsule-LPI Contains: S1. Dataset for Capsule-LPI; S2. The details of motifs; S3. The architecture of Capsule-LPI; S4. Features of LncADeep used in architecture comparison section; S5. Samples of the architectures of Capsule-LPI for different number of feature combination; S6. The potential lncRNA-disease association results. [file 12859_2021_4171_MOESM1_ESM.pdf]

Supplementary materials for Capsule-LPI

Supplementary catalog

S1. Dataset for Capsule-LPI 1
S2. The details of motifs 2
S3. The architecture of Capsule-LPI 3
S4. Features of LncADeep used in architecture comparison section 6
S5. Samples of the architectures of Capsule-LPI for different number of feature combination 6
S6. The potential lncRNA-disease association results 8

S1. Dataset for Capsule-LPI

Type	Source	Number
LncRNA-protein interaction pairs	NPInter database	6204
LncRNA-protein not interaction pairs	none	205,836
Sequence of lncRNA	NONCODE database	2356
Sequence of proteins	Uniprot database	90

LncRNA–protein interacting pairs are downloaded from NPInter database. It removes lncRNA–protein interacting pairs for non-human species as well as whose ncRNA is less than 200 nt in length. 6204 lncRNA–protein interacting pairs eventually retained. There are no negative samples of lncRNA–protein exists in the database, so we need to construct negative samples. The process of generating a negative sample set is as

follows: first, all the lncRNAs and proteins used in the positive sample are obtained from NPInter database, and there are 2356 lncRNAs and 90 proteins in total. Then the 2356 lncRNAs and 90 proteins are combined one by one, resulting in a total of 212,040 lncRNA-protein pairs. Finally, the 6204 lncRNA-protein pairs in the positive sample are removed, and 205,836 (21,040-6,204) lncRNA-protein pairs are considered as the negative samples. A large difference in the number of positive and negative samples can lead to prediction bias, so we randomly divided 205,836 negative samples into 33 sets, each set contains 6204 negative samples. Each lncRNA–protein non-interacting pairs set combines with lncRNA–protein interacting pairs set to train one model. Then we adopted EasyEnsemble [1] to ensembles 33 models to get the final model. 2356 lncRNAs' sequences and 90 proteins' sequences are included in the dataset. They are downloaded at NONCODE database and Uniprot database, respectively. The dataset can be downloaded at http://csbg-jlu.site/lpc/download/.

S2. The details of motifs

(a) Motifs of RNA

1. Fox1: UGCAUGU;
2. Nova: UCAUUUCAC, UCAUUUCAU, CCAUUUCAC, CCAUUUCAU;
3. Slm2: UAAAC, UAAAA, UAAUC, UAAUA;
4. Fusip1: AAAGA, AAAGG, AGAGA, AGAGG, CAAGA, CAAGG, CGAGA, CGAGG;
5. PTB: UUUUU, UUUCU, UCUUU, UCUCU;
6. ARE: UAUUUAUU;
7. hnRNPA1: UAGGGU, UAGGGA;
8. PUM: UGUAAAUA, UGUAGAUA, UGUAUAUA, UGUACAUA;
9. U1A: AUUGCAC;
10. HuD: UUAUUU;

11. QKI: AUUAAU, AUUAAC, ACUAAU, ACUAAC;

12. U2B: AUUGCAG;

13. SF1: UACUAAC;

14. HuR: UUUAUUU, UUUGUUU, UUUCUUU, UUUUUUU;

15. YB1: CCUGCG, UCUGCG;

16. {AU}: AU;

17. {UG}: UG.

18. Contains all motifs of Fox1, Nova, ARE, PUM and U1A

(b) Motifs of protein

1. {E}: E;

2. {H}: H;

3. {K}: K;

4. {R}: R;

5. {EE}: EE;

6. {KK}: KK;

7. {H, R}: H, R;

8. {HR, RH}: HR, RH;

9. {RS, SR}: RS, SR;

10. {RGG}: RGG;

11. {YGG}: YGG.

S3. The architecture of Capsule-LPI

(a) The first part are four fully connected layers using to learn sequence feature, motif information, physicochemical properties and secondary structure's high-level features separately.

For **sequence feature**, the architecture of fully connected layers is as follow:

**Layer**	**Nodes Number**	**Activation Function**	**Dropout**
Input layer	320		
1st hidden layer	200	PReLU	P=0.5
3rd hidden layer	100	PReLU	P=0.5
2nd hidden layer	50	PReLU	P=0.5
Output layer	3	tanh	

For **motif information**, the architecture of fully connected layers is as follow:

**Layer**	**Nodes Number**	**Activation Function**	**Dropout**
Input layer	29		
1st hidden layer	30	PReLU	P=0.5
3rd hidden layer	30	PReLU	P=0.5
2nd hidden layer	30	PReLU	P=0.5
Output layer	3	tanh	

For **physicochemical properties**, the architecture of fully connected layers is as follow:

**Layer**	**Nodes Number**	**Activation Function**	**Dropout**
Input layer	100		
1st hidden layer	100	PReLU	P=0.5
3rd hidden layer	50	PReLU	P=0.5
2nd hidden layer	20	PReLU	P=0.5
Output layer	3	tanh	

For **secondary structure**, the architecture of fully connected layers is as follow:

Layer	Nodes Number	Activation Function	Dropout
Input layer	20		
1st hidden layer	30	PReLU	P=0.5
3rd hidden layer	30	PReLU	P=0.5
2nd hidden layer	30	PReLU	P=0.5
Output layer	3	tanh	

We use 3 hidden layers for each fully-connected-layers, because the prediction accuracy does not growth significantly when the hidden layer number is bigger than 3, and a larger hidden layer number brings more computation. The number of neurons in each hidden layer was obtained through multiple experiments. The output dimension of each fully connected layers is 3, also be obtained by multiple experiments. PReLU has been used as activation function. To prevent overfitting, we added dropout layers to the hidden layers.

(b) The second part is capsule sub-network. Use capsule network to combine four feature vectors. Feature vectors are been treated as capsules and input to the capsule network. The capsule network outputs a vector of dimension 3, and the length of the vector represents the predicted outcome.

The architecture of capsule network is as follow:

Input capsules' number	Input capsules' dimension	Output capsules' number	Output capsules' dimension
4	3	1	3

In the capsule network section, the parameters to be learned come from only four transformation matrices, each with 3*3 = 9 parameters (Input capsules' dimension*

Output capsules' dimension). Total 36 parameters. Due to the small number of parameters, there is no overfitting and measures to prevent overfitting are not required.

The source code of the Capsule-LPI is available for download at http://csbg-jlu.site/lpc/download/.

S4. Features of LncADeep used in architecture comparison section

(a) Sequence features:

lncRNA: EDP of 4-mers, Fickett nucleotide feature, the EDP of the LCDS, LCDS length and coverage and mean hexamer score.

Protein: EDP of 3-mers.

(b) Structure features:

lncRNA: secondary structure, hydrogen-bonding and Van der Waals propensities.

Protein: secondary structure, hydrogen-bonding and Van der Waals propensities.

More details of each feature, refer to LncADeep [2].

S5. Samples of the architectures of Capsule-LPI for different number of feature combination

In the evaluation of different feature combinations section, we only adopt the architecture of Capsule-LPI. According to one, two and three kinds of features used for evaluation, the numbers of feature learning module in Capsule-LPI will be changed into one, two and three, respectively, And the number of capsules entered into the capsule network will be adjusted accordingly. The following figures are provided to further explain the detail, where the corresponding architectures for one (SeqFea), two (SeqFea, Motif) and three (SeqFea, Motif and PhysChem) are shown:

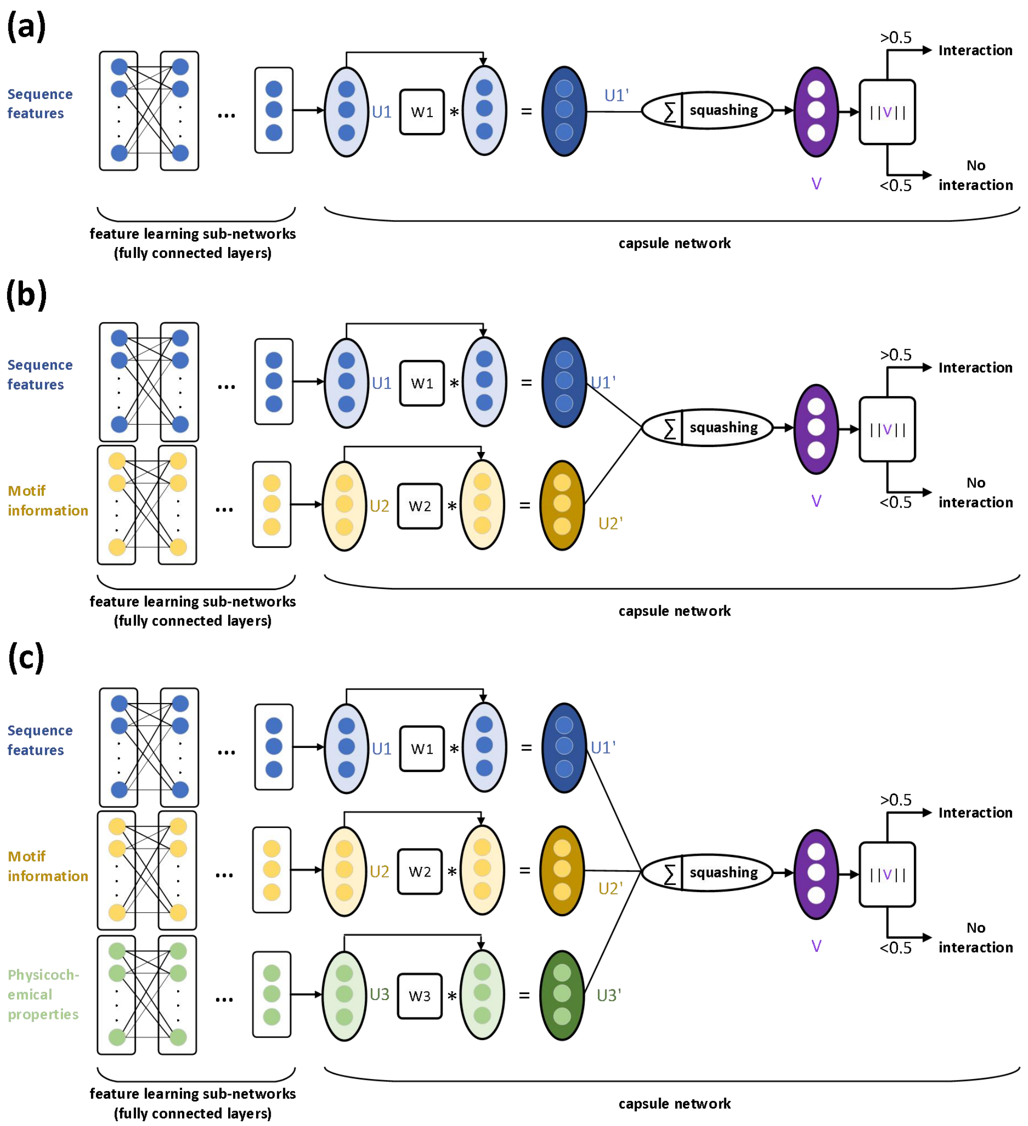

Additionally, it is easier to be performed in the source code shown as follows:

1. When defining the architecture, determine the types of features to use. In the source code, it is expressed as: "net = CLPI (SeqFea =False, Motif =True, PhysChem =True, SS =True)".

2. When training or forecasting a network, you only need to enter the features that you use. In the source code, it is expressed as: "test_probs = net (Motif =mot, PhysChem =pc, SS =ss)

By using Capsule-LPI in the above way, we can evaluate different features combinations.

S6. The potential lncRNA-disease association results

The potential disease association of each lncRNA is inferred by the enrichment analysis of disease association for the interacting proteins. Here we show the potential disease association results of the top 10 lncRNAs of interest on PubMed.

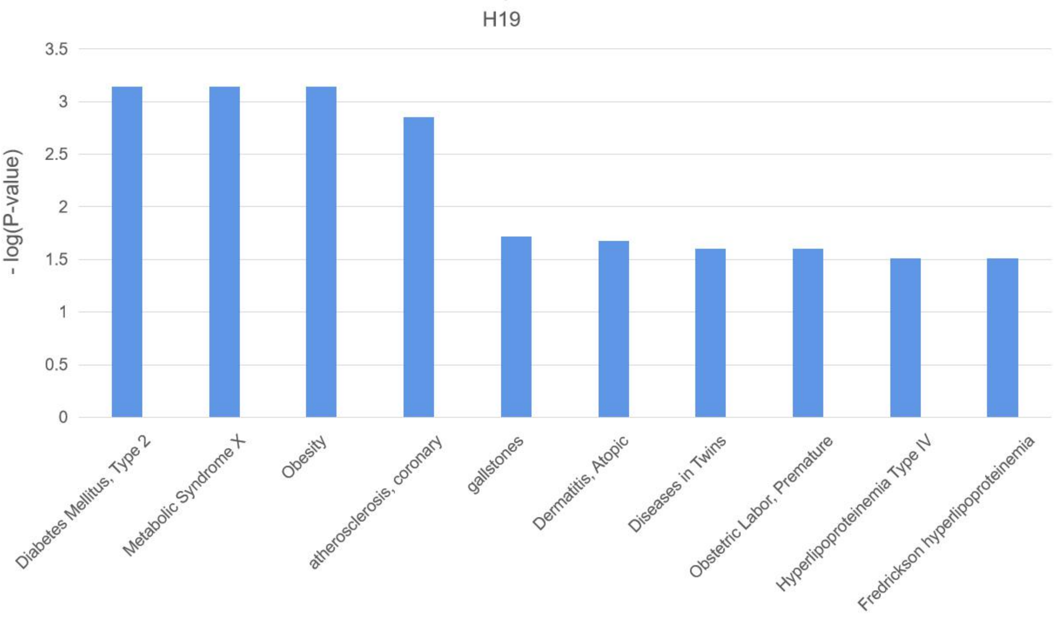

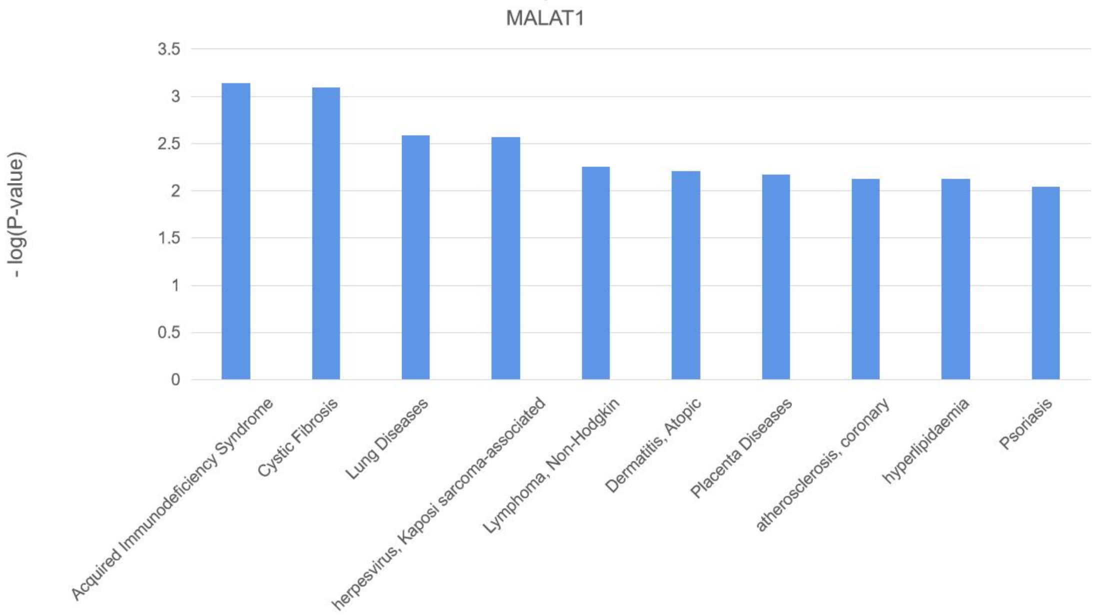

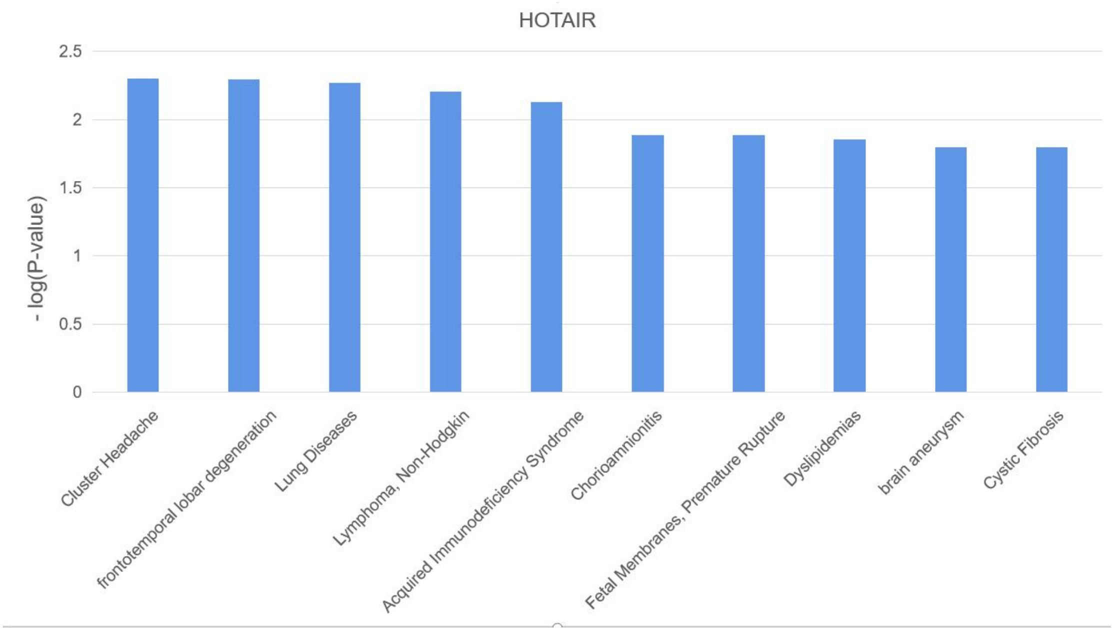
HOTAIR
- log(P-value)
2.5
2
1.5
1
0.5
0
Cluster Headache
frontotemporal lobar degeneration
Lung Diseases
Lymphoma, Non-Hodgkin
Acquired Immunodeficiency Syndrome
Chorioamnionitis
Fetal Membranes, Premature Rupture
Dyslipidemias
brain aneurysm
Cystic Fibrosis

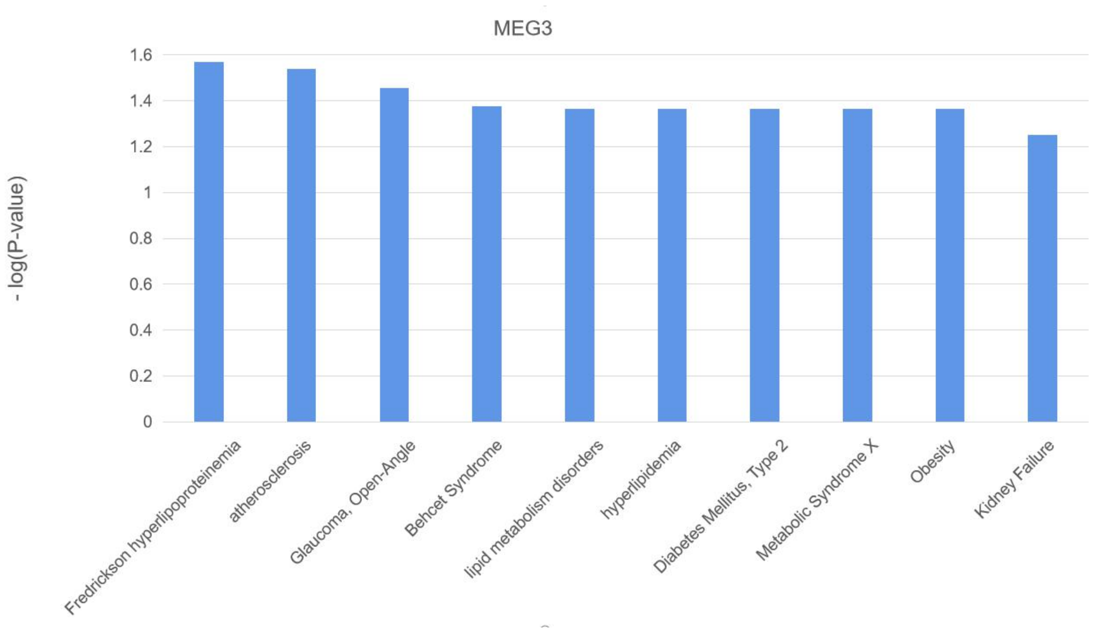
MEG3
- log(P-value)
1.6
1.4
1.2
1
0.8
0.6
0.4
0.2
0
Fredrickson hyperlipoproteinemia
atherosclerosis
Glaucoma, Open-Angle
Behcet Syndrome
lipid metabolism disorders
hyperlipidemia
Diabetes Mellitus, Type 2
Metabolic Syndrome X
Obesity
Kidney Failure

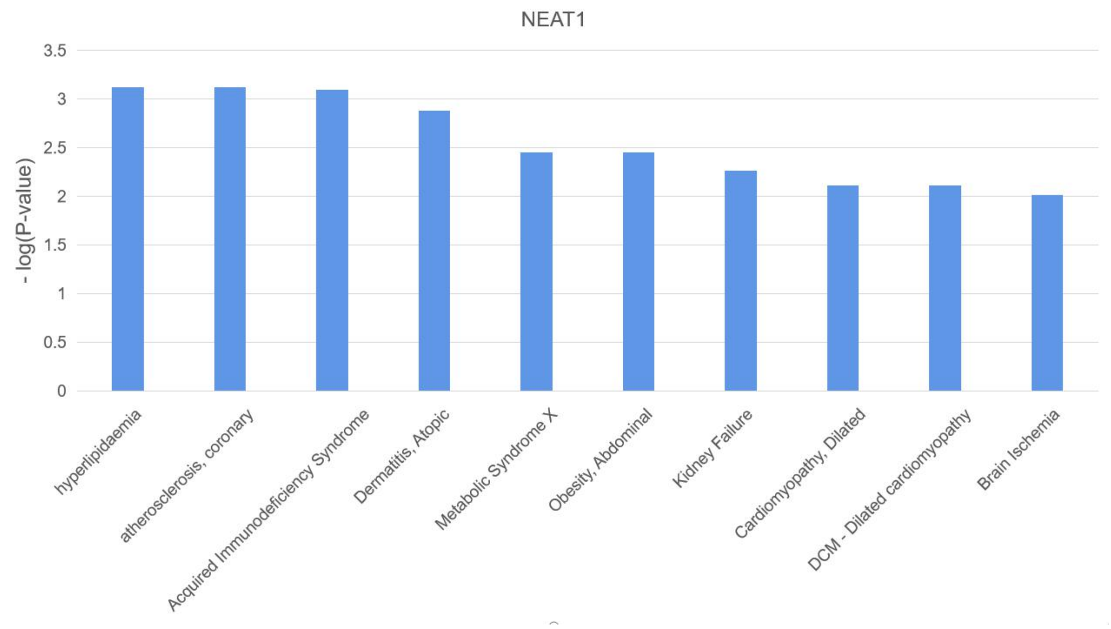
NEAT1
3.5
3
2.5
2
1.5
1
0.5
0
- log(P-value)
hyperlipidaemia
atherosclerosis, coronary
Acquired Immunodeficiency Syndrome
Dermatitis, Atopic
Metabolic Syndrome X
Obesity, Abdominal
Kidney Failure
Cardiomyopathy, Dilated
DCM - Dilated cardiomyopathy
Brain Ischemia

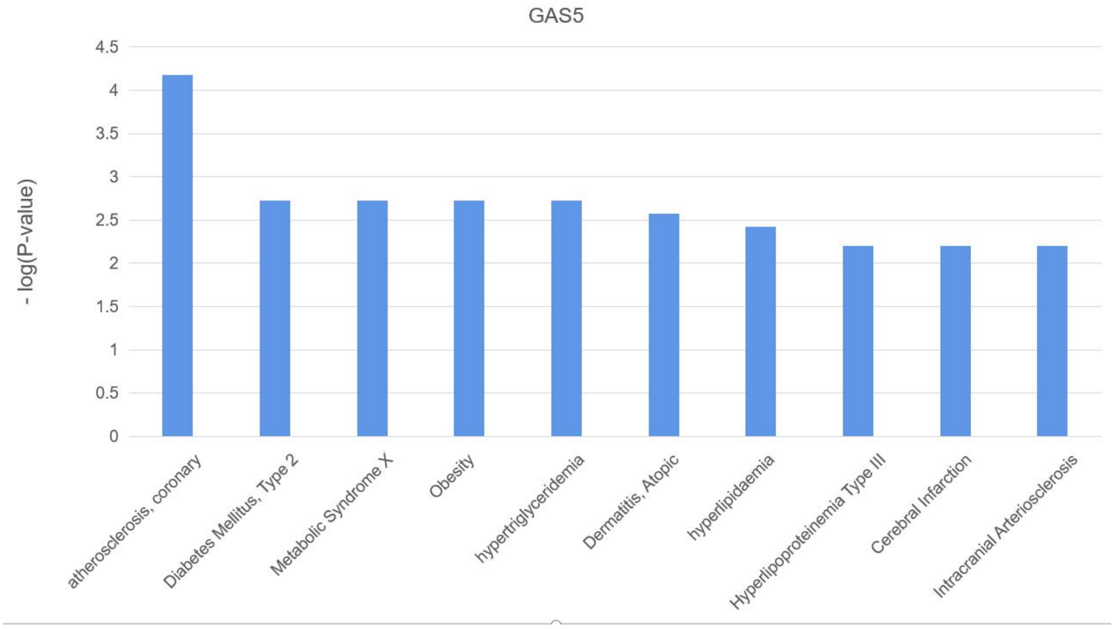
GAS5
4.5
4
3.5
3
2.5
2
1.5
1
0.5
0
- log(P-value)
atherosclerosis, coronary
Diabetes Mellitus, Type 2
Metabolic Syndrome X
Obesity
hypertriglyceridemia
Dermatitis, Atopic
hyperlipidaemia
Hyperlipoproteinemia Type III
Cerebral Infarction
Intracranial Arteriosclerosis

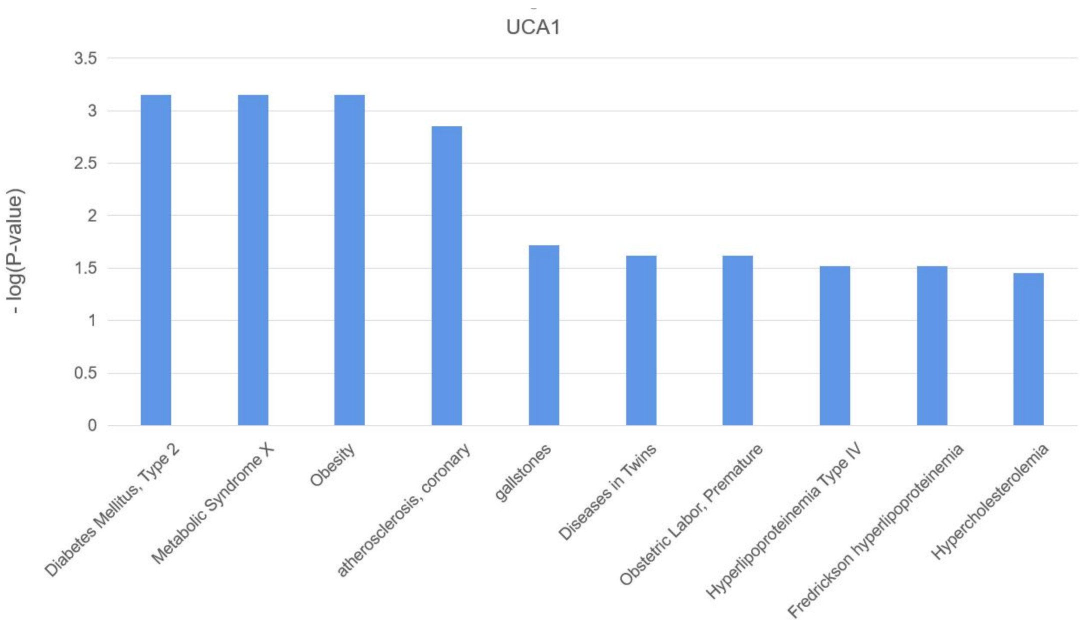
UCA1
- log(P-value)
3.5
3
2.5
2
1.5
1
0.5
0
Diabetes Mellitus, Type 2
Metabolic Syndrome X
Obesity
atherosclerosis, coronary
gallstones
Diseases in Twins
Obstetric Labor, Premature
Hyperlipoproteinemia Type IV
Fredrickson hyperlipoproteinemia
Hypercholesterolemia

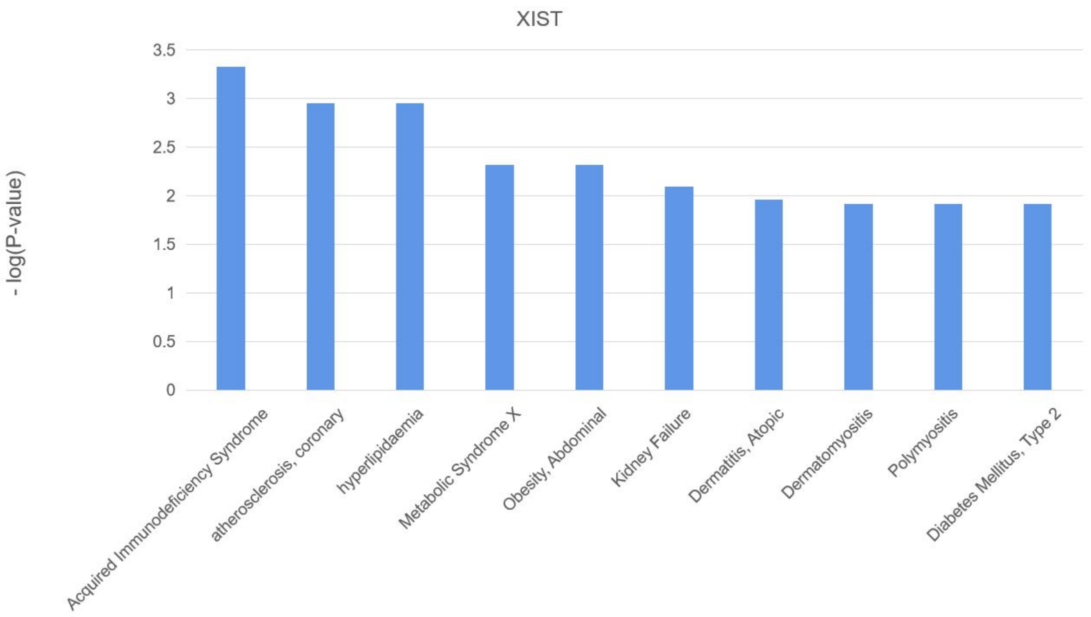
XIST
- log(P-value)
3.5
3
2.5
2
1.5
1
0.5
0
Acquired Immunodeficiency Syndrome
atherosclerosis, coronary
hyperlipidaemia
Metabolic Syndrome X
Obesity, Abdominal
Kidney Failure
Dermatitis, Atopic
Dermatomyositis
Polymyositis
Diabetes Mellitus, Type 2

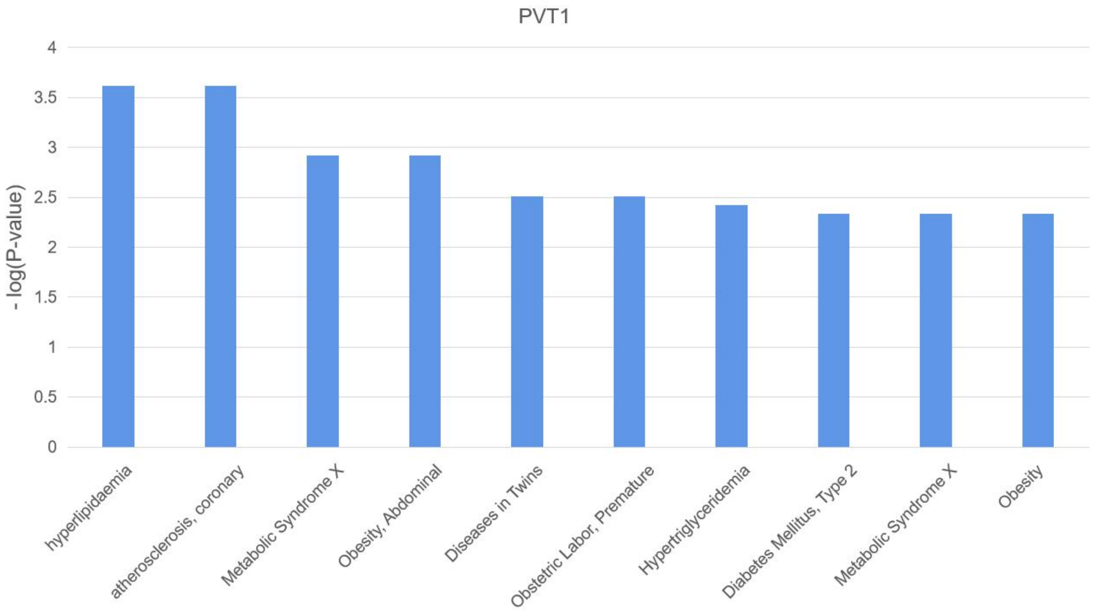
PVT1
- log(P-value)
0
0.5
1
1.5
2
2.5
3
3.5
4
hyperlipidaemia
atherosclerosis, coronary
Metabolic Syndrome X
Obesity, Abdominal
Diseases in Twins
Obstetric Labor, Premature
Hypertriglyceridemia
Diabetes Mellitus, Type 2
Metabolic Syndrome X
Obesity

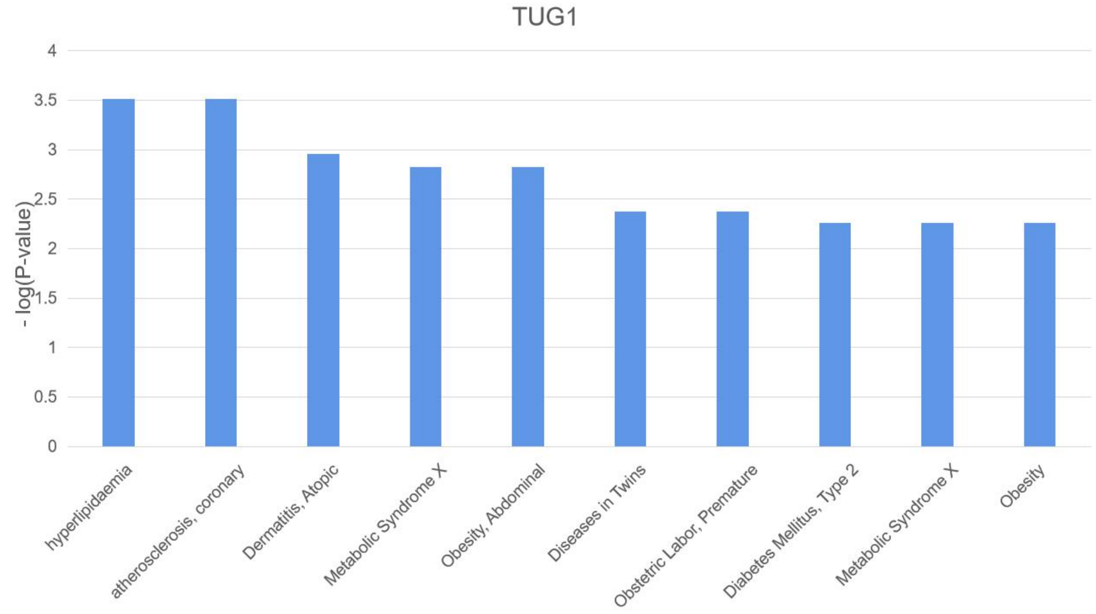
TUG1
- log(P-value)
0
0.5
1
1.5
2
2.5
3
3.5
4
hyperlipidaemia
atherosclerosis, coronary
Dermatitis, Atopic
Metabolic Syndrome X
Obesity, Abdominal
Diseases in Twins
Obstetric Labor, Premature
Diabetes Mellitus, Type 2
Metabolic Syndrome X
Obesity

References

1. Liu XY, Wu J, Zhou ZHJIToS, Man,, Cybernetics PBC: **Exploratory undersampling for class-imbalance learning**. 2009, **39**.
2. Cheng Y, Yang L, Man Z, Xie H, Zhang C, Wang MD, Zhu HJB: **LncADeep: An ab initio lncRNA identification and functional annotation tool based on deep learning**. 2018(22):22.
